# Liver MR relaxometry at 3T – segmental normal T_1_ and T_2_* values in patients without focal or diffuse liver disease and in patients with increased liver fat and elevated liver stiffness

**DOI:** 10.1038/s41598-019-44377-y

**Published:** 2019-05-30

**Authors:** V. C. Obmann, N. Mertineit, C. Marx, A. Berzigotti, L. Ebner, J. T. Heverhagen, A. Christe, A. T. Huber

**Affiliations:** 1 Department of Diagnostic, Interventional and Pediatric Radiology, Inselspital, Bern University Hospital, University of Bern, INO B, Freiburgstrasse 10, 3010 Bern, Switzerland; 2 Hepatology, Department of Visceral Surgery and Medicine, Inselspital, Bern University Hospital, University of Bern, INO A, Freiburgstrasse 10, 3010 Bern, Switzerland

**Keywords:** Liver fibrosis, Magnetic resonance imaging, Risk factors

## Abstract

Magnetic resonance (MR) T_1_ and T_2_* mapping allows quantification of liver relaxation times for non-invasive characterization of diffuse liver disease. We hypothesized that liver relaxation times are not only influenced by liver fibrosis, inflammation and fat, but also by air in liver segments adjacent to the lung – especially in MR imaging at 3T. A total of 161 study participants were recruited, while 6 patients had to be excluded due to claustrophobia or technically uninterpretable MR elastography. Resulting study population consisted of 12 healthy volunteers and 143 patients who prospectively underwent multiparametric MR imaging at 3T. Of those 143 patients, 79 had normal liver stiffness in MR elastography (shear modulus <2.8 kPa, indicating absence of fibrosis) and normal proton density fat fraction (PDFF < 10%, indicating absence of steatosis), defined as reference population. T_1_ relaxation times in these patients were significantly shorter in liver segments adjacent to the lung than in those not adjacent to the lung (p < 0.001, mean of differences 33 ms). In liver segments not adjacent to the lung, T_1_ allowed to differentiate significantly between the reference population and patients with steatosis and/or fibrosis (p ≤ 0.011), while there was no significant difference of T_1_ between the reference population and healthy volunteers. In conclusion, we propose to measure T_1_ relaxation times in liver segments not adjacent to the lung. Otherwise, we recommend taking into account slightly shorter T_1_ values in liver segments adjacent to the lung.

## Introduction

Magnetic resonance (MR) imaging of the liver is a powerful tool in diagnosis of focal liver disease and is frequently used in clinical routine^1,2^. Most conventional liver MR sequences measure relative signal intensities and therefore allow relative comparison between focal disease and adjacent normal hepatic parenchyma^3^. However, detection and quantification of diffuse liver disease remains challenging^4^ but is desirable to avoid invasive and expensive biopsies^5^.

Currently, existing non-invasive imaging biomarkers for diffuse liver disease include proton density fat fraction (PDFF) calculation^6^ and MR elastography^7,8^. Another emerging technique is the quantification of T_1_ relaxation time on parametric maps, which is routinely used in cardiac imaging^9^, for example in diffuse cardiac fibrosis^10,11^ or myocarditis^12^. Recently, the modified Look-Locker inversion recovery sequence (MOLLI) demonstrated great potential for application in diffuse liver disease^13,14^.

However, as known from cardiac applications, reference values should be established for different manufacturers, technical parameters and field strengths before using T_1_ mapping in clinical routine^15,16^. Further, it is known from cardiac imaging that T_1_ values might be influenced if measured in the ventricle close to the lung, why usually measurements in the septum are preferred^17^. Therefore, specific anatomical conditions of the liver with adjacent air-containing lungs and potential internal confounders, such as hepatic fat and iron composition, should be considered in hepatic T_1_ mapping^2^.

We hypothesized that T_1_ relaxation time is significantly shorter in liver segments adjacent to the lung than in liver segments not adjacent to the lung due to air-induced susceptibility effects at 3T.

## Results

### Patient characteristics

Patient characteristics are shown in Table 1. Patients without steatosis but with increased liver stiffness (shear modulus ≥ 2.8 kPa) showed a male predominance (78% males vs. 43% males in patients without fibrosis), a higher prevalence of diabetes (35% vs. 3%, p < 0.001), elevated GGT (110 ± 143 vs. 30 ± 33, p < 0.001), and prolonged extrinsic coagulation times (Quick 81 ± 20% vs. 99 ± 3%, p < 0.001). There was a tendency for more tobacco smokers in the group with increased liver stiffness, without statistical significance (p = 0.091). None of the patients was taking empagliflozin and only one patient was taking ezetimibe, both known to reduce liver fat as measured with PDFF^18,19^.

**Table 1:** Patient characteristics of the MR elastography study population (patients n = 143, volunteers n = 12, total n = 155).

	Reference population (n = 79) No steatosis (PDFF < 10%) Normal liver stiffness (shear modulus ≤ 2.8 kPa)	Positive Controls 1(n = 23)No steatosis (PDFF < 10%) Increased liver stiffness (shear modulus ≥ 2.8 kPa)	p - value	Positive Controls 2 (n = 26) Steatosis (PDFF > 10%) Normal liver stiffness (shear modulus ≤ 2.8 kPa)	Positive Controls 3 (n = 15) Steatosis (PDFF > 10%) Increased liver stiffness (shear modulus ≥ 2.8 kPa)	p - value	Negative Controls (n = 12) Healthy Volunteers
Age, years	51 ± 14	59 ± 13	0.023	56 ± 11	57 ± 12	0.925	31 ± 9
Male, %	34 (43%)	18 (78%)	0.004	15 (58%)	13 (87%)	0.084	7 (58%)
BMI, kg/m^2^	26 ± 8	27 ± 6	0.039	29 ± 5**	31 ± 7	0.559	22 ± 2
Tobacco	14 (18%)	8 (35%)	0.091	2 (8%)	7 (47%)	0.006	0 (0%)
Arterial hypertension	14 (18%)	7 (30%)	0.241	7 (27%)	5 (33%)	0.730	0 (0%)
Dyslipidaemia	8 (10%)	5 (22%)	0.162	0 (0%)	3 (20%)	0.043	0 (0%)
Diabetes Type 2	2 (3%)	8 (35%)	<0.001	3 (12%)	2 (13%)	>0.999	0 (0%)
Chronic renal insufficiency	1 (1%)	1 (4%)	0.402	0 (0%)	0 (0%)	>0.999	0 (0%)
≥1 medicament daily	20 (25%)	12 (52%)	0.021	6 (23%)	6 (40%)	0.300	0 (20%)
≥2 medicaments daily	5 (6%)	8 (35%)	0.001	2 (8%)	4 (27%)	0.168	0 (0%)
ASAT, U/l	24 ± 9	39 ± 23	0.029	29 ± 22	47 ± 30	0.041	N/A
ALAT, U/l	30 ± 41	36 ± 27	0.003	34 ± 21	55 ± 36	0.092	N/A
GGT, U/l	30 ± 33	110 ± 143	<0.001	37 ± 22	75 ± 67	0.224	N/A
Alkaline phosphatase, U/l	73 ± 36	87 ± 46	0.107	78 ± 19	88 ± 57	0.745	N/A
Bilirubin, μmol/l	10 ± 7	19 ± 17	0.016	8 ± 3	18 ± 15	0.281	N/A
Albumin	37 ± 3	35 ± 4	0.304	29 ± 15	34 ± 5	0.902	N/A
Quick, %	99 ± 3	81 ± 20	<0.001	95 ± 8	85 ± 23	0.601	N/A
APRI	0.73 ± 1.61	1.12 ± 1.00	0.039	0.67 ± 0.74	0.87 ± 1.15	>0.999	N/A
Creatinine, μmol/l	78 ± 22	76 ± 17	0.792	84 ± 21	82 ± 19	0.799	N/A
Combined: Diabetes, Dyslipidaemia, BMI > 25	1 (1%)	2 (9%)	0.127	0 (0%)	2 (13%)	0.128	N/A
Combined: Smoking, Diabetes, Dyslipidaemia, BMI > 25	1 (1%)	1 (4%)	0.402	0 (0%)	2 (13%)	0.128	N/A

Values represent the mean ± SD or n. P-values were calculated using the Mann-Whitney U or Fisher’s exact test, as appropriate. Comparisons between the two patient groups with steatosis and the reference group in the first column are indicated with *p < 0.05; **p < 0.001.

MR = magnetic resonance; PDFF = proton density fat fraction; BMI = body mass index; ASAT = aspartate aminotransferase; ALAT = alanine aminotransferase; GGT = gamma-glutamyltransferase; APRI = aspartate aminotransferase-to-platelet ratio index.

### Image quality

Out of 143 patients and 12 volunteers, resulting in 155 participants, there was at least one segment of good diagnostic image quality in 152/155 participants (98%) for T_1_ and in 149/155 participants (96%) for T_2_* (Table 2). In the right liver, segments V-VIII were measurable in at least 90% of the patients for T_1_. For T_2_*, the left liver segments were just measurable in 21–40%, while on the right side, the cranial right segments (VII and VIII) were measurable in 61–63% of patients and the right caudal segments (V and VI) were measurable in 86–88% of patients.

**Table 2:** Mapping quality in different liver segments in all study participants (patients n = 143, volunteers n = 12, total n = 155).

		Participants with good image quality	Segment VI/VII	p-value	Segment V/VIII	p-value	Segment IVa/b	p-value	Segment II/III	p-value
T_1_		152/155 (98%)								
Upper level	VII/VIII/IVa/II		140 (90%)	0.006	143 (92%)	0.031	135 (87%)	<0.001	134 (86%)	<0.001
Lower level	VI/V/IVb/III		140 (90%)	0.006	147 (95%)	0.218	102 (66%)	<0.001	88 (57%)	<0.001
T_2_*		149/155 (96%)								
Upper level	VII/VIII/IVa/II		95 (61%)	<0.001	97 (63%)	<0.001	62 (40%)	<0.001	58 (37%)	<0.001
Lower level	VI/V/IVb/III		137 (88%)	0.018	134 (86%)	0.004	58 (37%)	<0.001	33 (21%)	<0.001

Values represent the numbers of participant with liver segments with good image quality on T_1_ and T_2_* maps, respectively. Relative portion compared to total study population (n = 155) is shown with the % shown in brackets. The first column indicates the number of participants with at least one segment with good image quality, followed by the number of participants with good image quality maps in Segment VI/VII, V/VIII, Iva/B and II/III, respectively. In the upper part of the Table results for T_1_, in the lower part results for T_2_* are described.

P-values were calculated using Fisher’s exact test to compare the number of segments with good image quality at every localization to the number of at least one segment with good quality per patient.

### Location-based mapping results

**Figure 1:**
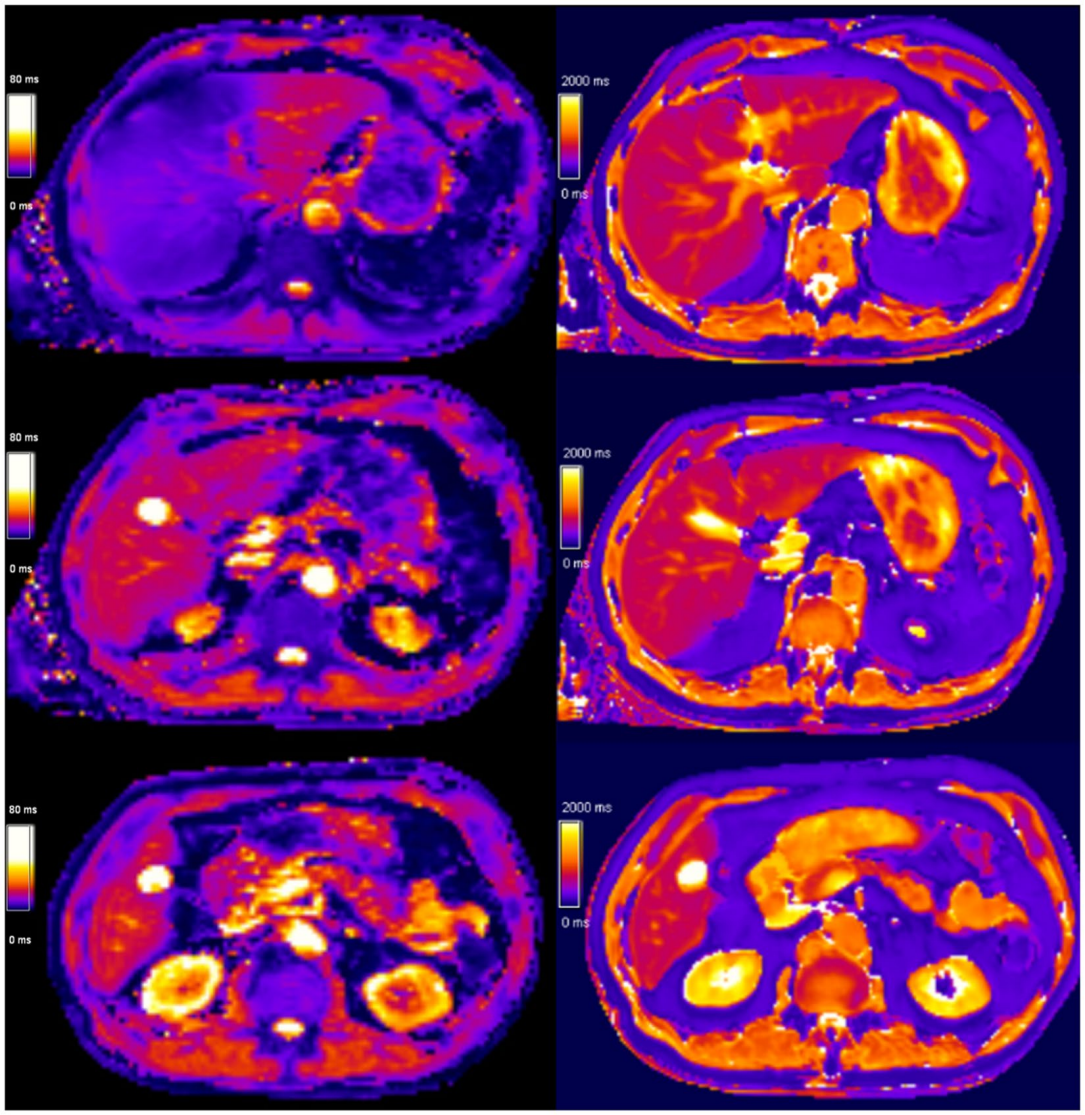
T_2_* and T_1_ mapping in a reference patient (male, 73 years old). Parametric maps with T_2_* on the left and T_1_ on the right are presented, from cranial (top) to caudal slices (bottom). Note the lower T_2_* values (blue) in cranial segments VII, VIII and IVa compared to the middle and lower slice (red) showing segments VI, V, IVb and III.

As shown in Table 3 and demonstrated in Fig. 1, T_1_ values were significantly lower in the cranial slices than in the caudal slices (mean of the differences 33 ms, p < 0.001). This did not count for the comparison between cranial segment II and caudal segment III, where no significant difference between both segments was detected (6 ms, p = 0.365). Values between segments were very comparable on each level, except for segment II, which showed values more comparable with the caudal level segments (Table 3). The same tendencies, but less pronounced, were observed for T_2_*.

**Table 3:** Reference values in different liver segments in patients with normal liver stiffness without steatosis (n = 79).

		All liver segments	N	Segment VI/VII	N	Segment V/VIII	N	Segment IVa/b	N	Segment II/III	N
T_1_	All segments	767 ± 82 ms	78								
Upper level	VII/VIII/IVa/II	751 ± 82 ms	78	742 ± 82 ms*	72	744 ± 84 ms*	76	752 ± 86 ms	72	770 ± 87 ms**	67
Lower level	VI/V/IVb/III	781 ± 84 ms	77	776 ± 85 ms*	73	785 ± 89 ms	76	775 ± 110 ms	61	776 ± 83 ms	43
Mean of the differences		33 ms		34 ms		42 ms		27 ms		6 ms	
P-value		<0.001		<0.001		<0.001		<0.001		0.365	
T_2_*	All segments	20 ± 5 ms	76								
Upper level	VII/VIII/IVa/II	19 ± 6 ms	60	18 ± 6 ms	50	18 ± 5 ms*	49	18 ± 6 ms	33	21 ± 7 ms**	32
Lower level	VI/V/IVb/III	20 ± 5 ms	75	20 ± 5 ms	68	20 ± 5 ms	67	20 ± 6 ms	30	19 ± 6 ms	15
Mean of the differences		0.9 ms		1.7 ms		1.6 ms		0.6 ms		−0.9 ms	
P-value		0.013		<0.001		<0.001		0.567		0.082	

Values represent the mean ± SD or n. P-values were calculated using a paired Mann-Whitney U test to compare upper and lower levels and each liver segment with the mean value of all liver segments of the same level: *p < 0.05; **p < 0.001.

Normal T_1_ values in reference patients were as follows: T_1_ = 780 ± 83 ms (segments not adjacent to the lung) and 746 ± 81 ms (segments adjacent to the lung) (Table 4). There were no significant differences between the younger healthy volunteers and the reference population (p = 0.358 for T_1_ and p = 0.521 for T_2_*).

**Table 4:** T_1_ values of lung-adjacent and non-lung-adjacent liver segments in all study participants (patients n = 143, volunteers n = 12, total n = 155).

		Normal liver stiffness (shear modulus <2.8 kPa)	N	p-value	Negative controls			Positive controls					
					Healthy volunteers	N	p-value	Increased liver stiffness (shear modulus ≥2.8 kPa)	N	p-value	Significantly increasing liver stiffness (shear modulus ≥3.5 kPa)	N	p-value
**No Steatosis**													
T_1_ adjacent to the lung	VII/VIII/IVa	746 ± 81 ms	78		764 ± 56 ms	12	0.291*	835 ± 106 ms	21	<0.001*	885 ± 98 ms	13	<0.001*
T_1_ not adjacent to the lung	VI/V/IVb/II/III	780 ± 83 ms	78		799 ± 59 ms	12	0.358*	849 ± 107 ms	23	0.011*	910 ± 98 ms	13	<0.001*
**Steatosis**													
T_1_ adjacent to the lung	VII/VIII/IVa	809 ± 100 ms	24	0.009*				843 ± 141 ms	15	0.544**	906 ± 46 ms	6	0.013**
T_1_ not adjacent to the lung	VI/V/IVb/II/III	833 ± 95 ms	24	0.019*				889 ± 148 ms	14	0.269**	932 ± 41 ms	6	0.013**

Values represent the mean ± SD or n. P-values were calculated using the Mann-Whitney U test, *compared to patients with normal liver stiffness without steatosis, **compared to patients with normal liver stiffness with steatosis.

**Figure 2:**
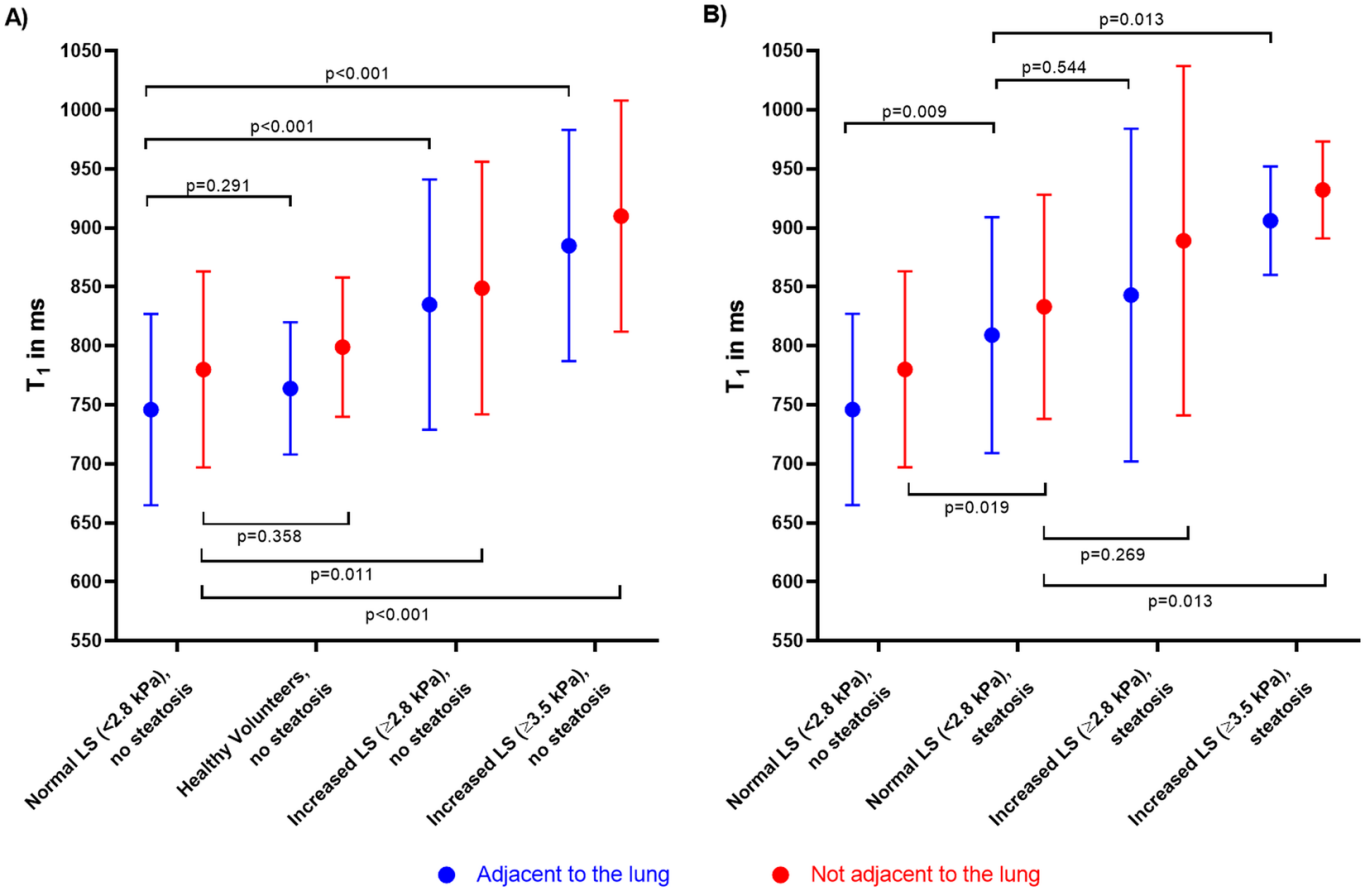
T_1_ values of liver segments adjacent to the lung and liver segments not adjacent to the lung in different patient groups. T_1_ values are illustrated as mean and standard deviation in ms for liver segments adjacent to the lung in blue and for liver segments not adjacent to the lung in red. Panel (A) compares all participants without liver steatosis: reference population (normal liver stiffness, no steatosis), healthy volunteers, increased liver stiffness (LS) ≥ 2.8 kPa and ≥3.5 kPa. In panel (B) T_1_ values of the reference population are compared with patients with liver steatosis and normal LS (<2.8 kPa) and increased LS (≥2.8 and ≥3.5 kPa). P-values were calculated using the Mann-Whitney U test. LS = liver stiffness.

In positive patients with steatosis as well as in patients with increased liver stiffness, T_1_ relaxation time was significantly longer than in the reference population (p = 0.019 and p = 0.011 and p < 0.001 for steatosis only, increased stiffness ≥ 2.8 kPa only and increased stiffness ≥ 3.5 kPa only, respectively). These results are illustrated in Fig. 2.

### Multivariate analysis

**Figure 3:**
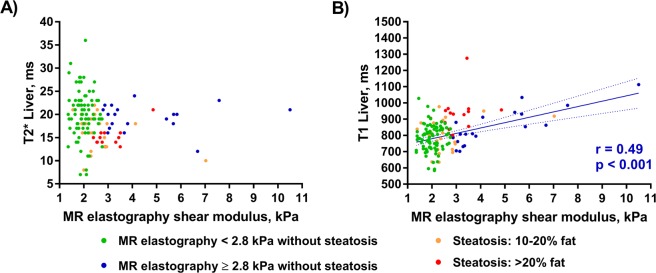
Correlation among MR elastography and T_1_ and T_2_* values. Subgroups are color-encoded for better illustration. Measured liver stiffness without steatosis is demonstrated in green if <2.8 kPa and in blue if ≥2.8 kPa Patients with steatosis are shown in yellow (10–20% fat) and red (>20%), respectively. (**A**) Comparison of T_2_* and MR elastography without significant correlation in any group. (**B**) Comparison of T_1_ with MR elastography.

In multivariate analysis of patients and volunteers without focal or diffuse liver disease, T_2_* time was a significant confounder of T_1_ time (p < 10^−15^), while PDFF, age and sex (p = 0.249, 0.722, 0.687, respectively) were not (Fig. 3).

### Inter-reader reliability

The ICC values were excellent for both T_1_ and T_2_*, as measured in segment VI. The ICC was 0.97 (95%-CI: 0.91–0.99) for T_1_ and 0.91 (95%-CI: 0.78–0.97) for T_2_*.

## Discussion

This study shows that T_1_ relaxation time is significantly shorter in liver segments adjacent to the lung than in liver segments not adjacent to the lung. We calculated a mean T_1_ value of 780 ± 83 ms at 3T in liver segments not adjacent to the lung (segments II, III, IVb, V, VI), while T_1_ was around 30 ms shorter in liver segments adjacent to the lung (segments IVa, VII, VIII) in a patient population, without focal (based on CT) or diffuse (based on PDFF and MR elastography) liver disease.

**Figure 4:**
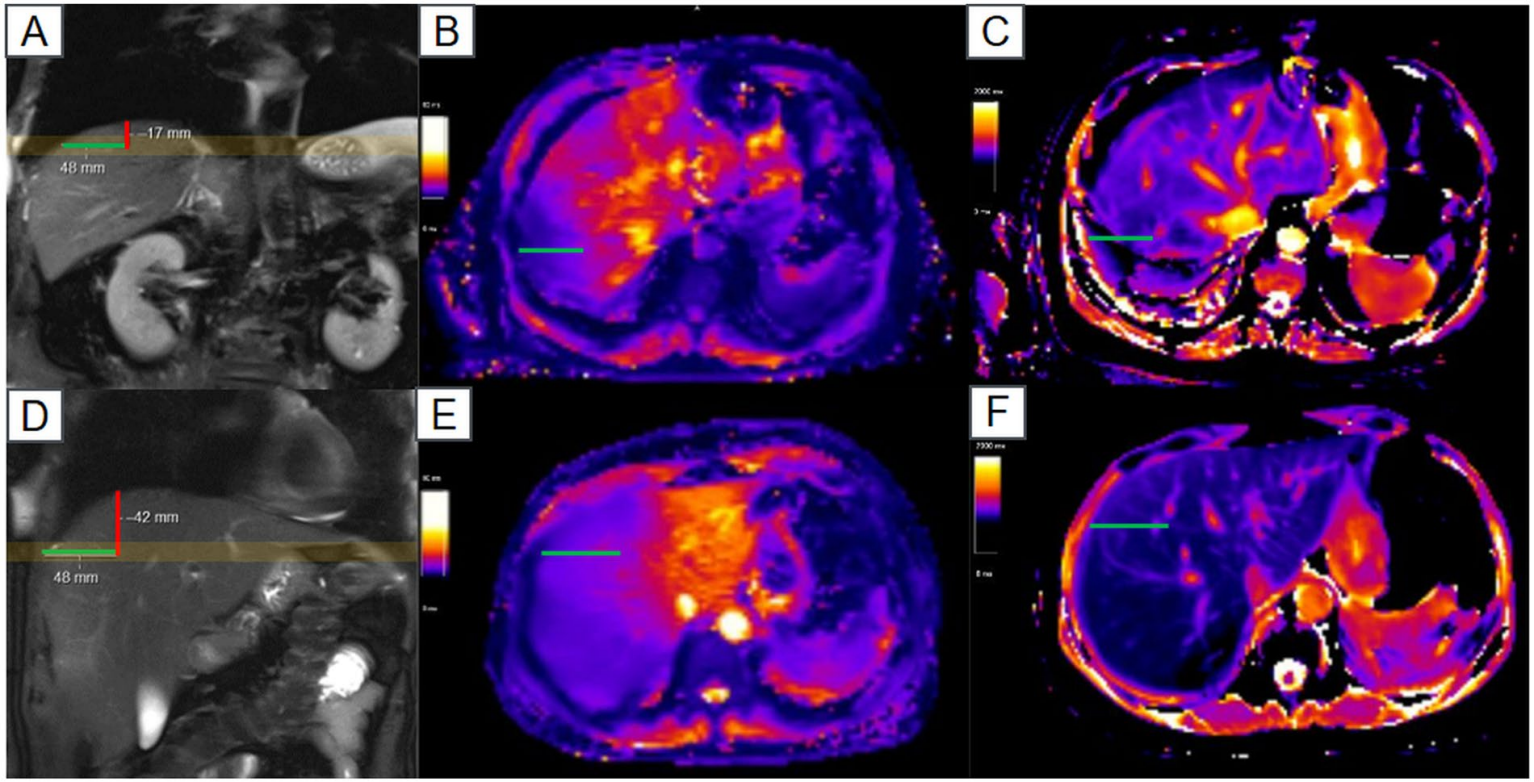
Localization of acquired maps. Correlation of T_2_* maps (**B,E**), T_1_ maps (**C,F**) and coronal T_2_w HASTE images (**A,D**) for two patients are shown. The yellow box in displays A and D indicate the location of the 10 mm thick mapping slices. The green lines show the area with lower relaxation times and their distance from the liver border. The red lines demonstrate the distance from there to the liver dome. In the patient in the upper row (**A–C**), a 67-year-old man, partial volume is a good explanation for shorter relaxation times with obvious artefacts in Segment VII on the T_1_map (**C**), while in the other patient (**D–F**), a 30-year-old women, shorter relaxation times cannot be explained by partial volume alone. Although patient might have shown different breathing between coronal and axial sequences, no significant difference in acquisition level is to assume since the stomach is still seen on axial slices.

This difference may be explained by different reasons. One possibility are susceptibility effects from adjacent air in the lungs. Susceptibility differences between liver and lungs cause off-resonance, which may lead to T_1_ underestimation. Similar effects are known from myocardial T_1_ mapping^20,21^. In addition, there is a T_2_-dependency of the MOLLI based T_1_ mapping sequence due to its bSSFP design, therefore influencing the measured T_1_ value. Another explanation might be partial volume effects at the liver dome since the slice thickness is 10 mm (Fig. 4), which is also known from neuroimaging^22^. However, the regions of interests (ROI) were drawn carefully to exclude liver vessels and outer 10 mm of the liver border, to prevent this possible bias as much as possible.

While differences between cranial and caudal segments in the right liver lobe and segment IVa vs. IVb were comparable, the T_1_ relaxation time difference between segment II and III was smaller. A potential explanation for this different behaviour might be that segment II is in close anatomical neighborhood to the heart and not to the lungs as the cranial segments VII, VIII and IVb are. We therefore propose to measure T_1_ relaxation time in liver segments V and VI with excellent inter-reader-reliability, when a representative T_1_ quantification of the liver is intended. These segments are normally targeted for liver biopsy and may be regarded as representative for the whole liver. However, for a more detailed segmental analysis of T_1_ relaxation times in the liver shorter normal T_1_ values in liver segments adjacent to the lung should be taken into account. T_2_* seems to be more prone to respiratory and pulsation artefacts from the beating heart, which might explain lower image quality in segments of the left liver lobe (adjacent to the heart) and in cranial segments, while image quality in caudal left segment V and VI showed the best image quality. Electrocardiography gating might improve image quality, which was not tested in this study.

There are very few published data on normal T_1_ values of the liver at 3T using the MOLLI technique. Katsube *et al*. presented a mean value of 836 ± 69 ms in 16 patients with normal liver function. The slightly lower values in our study (780 ± 83 ms in reference patients, 799 ± 59 ms in healthy volunteers) may be explained by a smaller population in the study of Katsube *et al*., as well as the by the fact that Katsube *et al*. defined the normal population based on normal liver function in laboratory tests, which does not exclude patients with steatosis or early liver fibrosis^23^. Other published normal T_1_ values were generated with other mapping techniques, but are nevertheless in a similar range, such as 824 ± 61 ms in 26 healthy volunteers using a spiral GRAPPA-based method^24^, 825 ± 49 ms in 6 healthy volunteers using an inversion recovery method with refocusing pulses^25^, and 745 ± 65 ms in 8 healthy volunteers using MR fingerprinting^26^. Slight differences might be explained by different techniques of assessment and small sample sizes used in the cited studies. This underlines the recommendation, that normative values should be established for the particular site and set-up for different manufacturers, technical parameters and field strengths before using T_1_ mapping in clinical routine^15,16^. In accordance with other published studies, increased T_1_ time correlated with increased liver stiffness in MR elastography as a non-invasive surrogate for liver fibrosis^2,13,14,23^. Yoon *et al*. published T_1_ values in patients with chronic liver disease (863 ± 81 ms) and Child A liver cirrhosis (879 ± 86 ms)^27^ in the same range as we present here (849 ± 107 ms for early liver fibrosis and 910 ± 98 ms for significant fibrosis). T_1_ values calculated with commercially available software solutions using the MOLLI mapping technique have been shown to correlate with the severity of NAFLD/NASH and fibrosis^28^ and may predict clinical outcome in these patients^29^. We demonstrated an increased T_1_ time in patients with steatosis, which is in accordance with other published studies^30^. This is a paradox, since fat has a much shorter T_1_ time than water. According to recent publications, off-resonance effects in imaging voxels containing a mixture of fat and water signals may explain this finding^31,32^.

In addition to fat and fibrosis content as well as susceptibility-effects in liver segments adjacent to the lung, there are several other potential confounders of T_1_ and T_2_*. One known confounder inducing T_1_ shortening is liver iron content^2,33^, however patients with iron overload have been excluded in this study. Liver blood distribution and oxygenation levels are might influence T_1_ and T_2_* times as well. These effects and possible influences on T_1_ and T_2_* need further investigation. In multivariate analysis of reference patients without focal or diffuse liver disease and healthy volunteers, we could not demonstrate a significant age- or sex- dependency of the measured T_1_ values. However, there was a collinearity between T_1_ and T_2_* values. Further research should be performed to show whether T_1_ is T_2_* dependent (e.g. due to fat, iron composition, blood distribution and eventually blood oxygenation influencing T_2_*) or whether it is the other way round and T_2_* is influenced by T_1_ due to a too short TR in the used T_2_*-mapping sequence. Also using a multiparametric approach combining T_1_, T_2_*, MRE and PDFF might deliver further insights.

Our study highlights the importance of standardized technical parameters and well-defined normal values when performing T_1_ mapping of the liver. For an accurate non-invasive characterization of diffuse liver disease, a combined analysis of different MR parameters such as T_1_ mapping, T_2_* mapping, elastography and PDFF should be performed. Further research will show how a multiparametric combination of those MR imaging biomarkers may help differentiating and quantifying diffuse liver disease.

### Limitations

Our study has several limitations. First, we focused on a population without known chronic liver disease. Due to the study design and associated ethical considerations, liver biopsy was not possible. Instead, we used MR elastography and PDFF, which have been shown to correlate very well with biopsy-confirmed fibrosis^34^ and steatosis grades^35^, as the non-invasive gold standard, and we included negative and positive controls based on these imaging techniques. Another limitation is that we did not obtain full 3D coverage of the liver, as we used commercially available Siemens MOLLI sequences with 2D acquisitions on three transverse slices. Nevertheless, we present a segmental comparison of T_1_ mapping in a reference population, as well as in negative and positive controls. Newer 3D mapping sequences or MR fingerprinting may offer even more applications for future use.

## Conclusion

When analysing T_1_ maps in the liver at 3T, we propose to measure T_1_ relaxation times in liver segments not adjacent to the lung. Otherwise, we recommend taking into account slightly shorter T_1_ normal values in liver segments adjacent to the lung.

## Methods

### Study population

This prospective cross-sectional study was approved by the institutional review board (Kantonale Ethikkomission Bern, IRB number 282–15) and was conducted in accordance with relevant guidelines and regulations after obtaining written patient informed consent. All participants underwent multiparametric MR imaging at 3T in our institution between 03/2016–06/2017, including T_1_ and T_2_* mapping, proton density fat fraction (PDFF) quantification and MR elastography.

**Figure 5:**
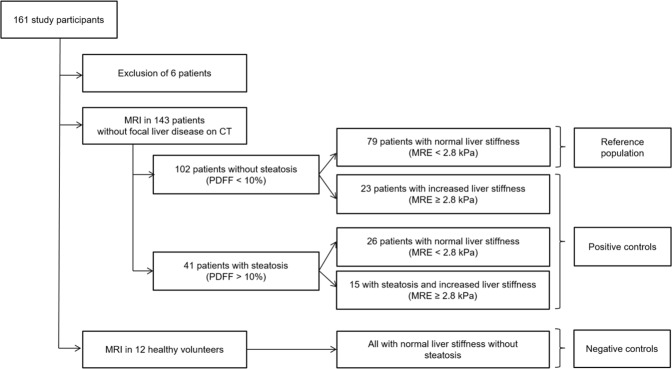
Study participant workflow. A total of 161 participants were recruited for the study. Six patients were excluded because of an aborted MRI scan due to claustrophobia (n = 1) or due to technically inadequate MRE scans (n = 5). Resulting included study population consisted of 12 healthy volunteers with normal liver stiffness and without steatosis (negative controls) and 143 patients without focal liver disease on CT. The MRI scans of 143 patients were included in statistical analysis. There were 102 patients who did not show steatosis, and 79 of these 102 patients also showed normal liver stiffness (reference patients). The remaining 23 showed increased liver stiffness (≥2.8 kPa). Forty-one patients had liver steatosis with PDFF > 10% (26 with normal liver stiffness, 15 with increased liver stiffness). Patients with increased liver stiffness and/or steatosis were defined as positive controls.

A total of 161 study participants were recruited, while 6 patients had to be excluded due an incomplete MR exam due to claustrophobia or technically uninterpretable MR elastography. Resulting study population consisted of twelve healthy volunteers without a history of liver disease (negative controls) and 143 patients (Fig. 5). The included 143 patients were selected based on acquired abdominal computed tomography (CT) scans without focal liver disease (cysts > 2 cm, solid lesion >1, prior liver surgery). Out of the 143 patients, we then defined a reference population (n = 79) without focal (based on CT) or diffuse liver disease (based on MR elastography shear modulus < 2.8 kPa and PDFF < 10%). CT scans in the reference population were performed with following indications: trauma (n = 14), abdominal pain (n = 19), infection (n = 23) and tumor search (n = 23). The remaining patients (n = 64) were assigned to positive control groups with diffuse liver disease (MR elastography shear modulus ≥2.8 kPa and/or PDFF ≥10%).

Clinical information and laboratory test results were recorded for the included patients. Clinical parameters included age, gender, body mass index (BMI), history of diabetes or hypertension, daily drug intake, tobacco use and alcohol consumption. Biological parameters included dyslipidemia, platelet count, quick value, total bilirubin levels, gamma-glutamyltranspeptidase (GGT), aspartateaminotransferase (AST), alanine aminotransferase (ALT), alkaline phosphatase, albumin, creatinine and hematocrit.

### Sample size estimation

To detect a mean difference in T_1_ of 5% (=40 ms) between the upper and lower liver segments with a significance level of 0.05 and a power of 0.8, a sample size of 72 was needed. A mean T_1_ time of 800 ms with standard deviation of 85 ms was based on a pilot readout for sample size calculations.

### MR imaging technique

Patients were examined with a 3T-MR system (Verio, Siemens Healthineers) in a fasting state (>6 h). For T_1_ mapping, we used an axial-acquired, MOLLI single breath-hold sequence (echo time (TE) of 1.01 ms, data acquisition window of 740 ms, inversion time (TI) 225 ms (3 inversion pulses, starting at 65 ms with an increment of 80 ms), flip angle (FA) 35°, 8-mm slice thickness, field-of-view (FOV) 384, matrix 154 × 192 pixels, and scan time of 11 s) with a 3-3-5 design (acquisition during 3 heartbeats, pause during 3 heartbeats for relaxation purposes and acquisition during another 5 heartbeats). T_2_* mapping was performed with a multiecho gradient echo (GRE) single breath-hold sequence (12 echoes with a TE between 0.93–14.2 ms, TR of 200 ms, FA 18°, FOV 400, 10-mm slice thickness, and scan time of 19 s). T_1_ and T_2_* maps were generated on three single slices in the upper, mid and lower liver. PDFF was calculated using the Dixon method with axial T_1_-weighted axial vibe images (TE of 2.45 ms and 3.68 ms, TR of 5.47 ms, FA 9°, 3-mm slice thickness, and scan time of 22 s) to differentiate patients with and without liver steatosis. For MR elastography, a pneumatic driver (Resoundant) was placed on the right upper quadrant transmitting shear waves by continuous acoustic vibrations with a frequency of 60 Hz. The liver shear stiffness in kPa in the right upper liver lobe was determined with a gradient echo-based elastography sequence (WIP package 622 provided by Siemens Healthineers, 3 single-slice acquisitions with 5-mm slice thicknesses) using the 95% confidence map of stiffness. A shear modulus ≥2.8 kPa was considered to represent early liver fibrosis (corresponding to histology fibrosis grade ≥F1, according to the Metavir staging system), while a shear modulus ≥3.5 kPa was defined as significant liver fibrosis (corresponding to histology fibrosis grade ≥F3)^8,36,37^.

### MR imaging analysis

**Figure 6:**
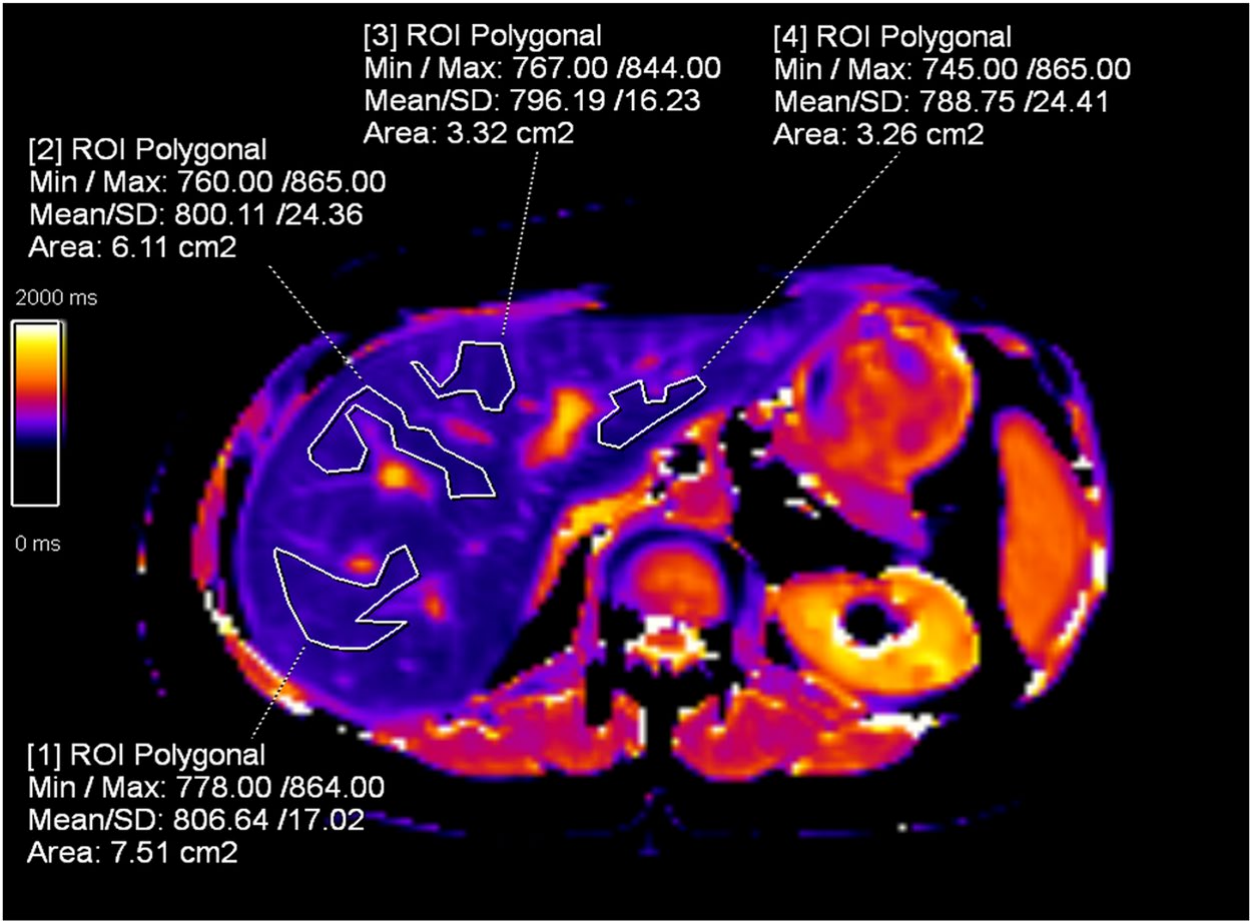
T_1_ map of a 49-year-old female patient with normal liver fat and stiffness. Polygonal ROI show normal T_1_ values in liver segments VII (ROI 1), VIII (ROI 2), IVa (ROI 3) and II (ROI 4). ROI were drawn with distance to the liver border and by avoiding large blood vessels and bile ducts. ROI = region of interest.

Prior to any measurements image quality on relaxometry maps was assessed by an experienced radiologist (V.O., 5 years of experience in hepatic imaging). For T_1_ and T_2_* mapping, 8 polygonal regions of interest (ROI) were drawn in liver segments II-VIII by the radiologist (V.O.) who was blinded to the patient’s clinical history. In liver segments without excellent image quality (e.g. due to motion artefacts) or in segments that were not captured on any of the three acquired slices, no ROI was drawn at this location, and thus no value was assigned to the respective segment. The mean ROI size was 685 ± 203 mm^2^. Large blood vessels, bile ducts and regions with partial volume, including air or perihepatic fat at the liver border, were excluded (Fig. 6). Another radiologist (A.H.), who was blinded to the patient’s clinical history and had 7 years of experience in hepatic imaging, measured the shear modulus (in kPa) on MR elastography images and the PDFF (in %) based on in- and out-of-phase DIXON images in the right liver on three slices, using polygonal ROIs to exclude vessels and partial volume at the liver borders. The median value of the three ROIs was then calculated.

### Statistical analysis

Analysis was performed with the statistical software package R (version 3.4.1, R Foundation for Statistical Computing)^38^ and GraphPad Prism (version 7.1, GraphPad Software Inc.). Clinical characteristics were compared between groups using the Wilcoxon test for continuous variables or Fisher’s exact test for categorical variables. The p-value for significance was <0.05. T_1_ and T_2_* mapping parameters with good quality were then compared between segments using a paired Wilcoxon test. To address intersegmental variability and to identify the best area for reference, the median values of the liver segments adjacent to the lung (VII, VIII, IVa) and those not adjacent to the lung (VI, V, IVb, II, III) were calculated for the reference population and compared with the negative and positive controls using the Wilcoxon test.

To assess possible confounders in the reference population and among healthy volunteers (n = 79 + 12), a multivariate regression model was used with T_1_ (segments not adjacent to the lung) as the outcome and sex (dummy-coded), age, PDFF and T_2_* (segments not adjacent to the lung) as variables. Age and sex were chosen as basic demographic characteristics that might bias the measured T_1_ in the liver while PDFF and T_2_* were added as known representatives of liver fat and iron content that might influence T_1_. Pearson correlation was used to compare MR elastography shear modulus with T_1_ values. For interrater reliability, T_1_ and T_2_* relaxation time was measured in segment VI in 20 randomly selected consecutive patients by a second radiologist (A.H.), who was blinded to the patient’s clinical history and had 7 years of experience in hepatic imaging. The two-way consistency intraclass correlation (ICC) was then calculated and classified as follows: ICC 0.4-0.59 defined as fair; 0.6–0.74 defined as good; and 0.75–1.00 defined as excellent^39^.

### Human subject research

This prospective cross-sectional study was approved by the institutional review board (IRB number 282-15) and was conducted after obtaining written patient informed consent.

## Data Availability

Data generated for analysis during this study are included in this published article. Original patient data files are precluded from dissemination following Swiss Federal Law regulations (https://www.admin.ch/opc/de/officialcompilation/2013/3381.pdf). Data requests may be sent to: Kantonale Ethikkommission für die Forschung Murtenstrasse 31, 3010 Bern (Tel. +41 31 633 70 70, Fax +41 31 633 70 71, info.kek.kapa@gef.be.ch).
